# Post-exposure prophylaxis vaccination rate and risk factors of human rabies in mainland China: a meta-analysis

**DOI:** 10.1017/S0950268818003175

**Published:** 2018-12-04

**Authors:** D. L. Wang, X. F. Zhang, H. Jin, X. Q. Cheng, C. X. Duan, X. C. Wang, C. J. Bao, M. H. Zhou, T. Ahmad

**Affiliations:** 1 Department of Epidemiology and Health Statistics, School of Public Health, Southeast University, Nanjing (210009), China; 2 Key Laboratory of Environmental Medicine Engineering, Ministry of Education, School of Public Health, Southeast University, Nanjing (210009), China; 3 Jiangsu Provincial Center for Disease Control and Prevention, China

**Keywords:** Meta-analysis, PEP vaccination rate, rabies, risk factors

## Abstract

Rabies is one of the major public health problems in China, and the mortality rate of rabies remains the highest among all notifiable infectious diseases. A meta-analysis was conducted to investigate the post-exposure prophylaxis (PEP) vaccination rate and risk factors for human rabies in mainland China. The PubMed, Web of Science, Chinese National Knowledge Infrastructure, Chinese Science and Technology Periodical and Wanfang databases were searched for articles on rabies vaccination status (published between 2007 and 2017). In total, 10 174 human rabies cases from 136 studies were included in this meta-analysis. Approximately 97.2% (95% confidence interval (CI) 95.1–98.7%) of rabies cases occurred in rural areas and 72.6% (95% CI 70.0–75.1%) occurred in farmers. Overall, the vaccination rate in the reported human rabies cases was 15.4% (95% CI 13.7–17.4%). However, among vaccinated individuals, 85.5% (95% CI 79.8%–83.4%) did not complete the vaccination regimen. In a subgroup analysis, the PEP vaccination rate in the eastern region (18.8%, 95% CI 15.9–22.1%) was higher than that in the western region (13.3%, 95% CI 11.1–15.8%) and this rate decreased after 2007. Approximately 68.9% (95% CI 63.6–73.8%) of rabies cases experienced category-III exposures, but their PEP vaccination rate was 27.0% (95% CI 14.4–44.9%) and only 6.1% (95% CI 4.4–8.4%) received rabies immunoglobulin. Together, these results suggested that the PEP vaccination rate among human rabies cases was low in mainland China. Therefore, standardised treatment and vaccination programs of dog bites need to be further strengthened, particularly in rural areas.

## Introduction

Rabies is a zoonotic disease caused by the rabies virus, which belongs to the family Rhabdoviridae and genus *Lyssavirus*. Rabies is estimated to be responsible for approximately 60 000 human deaths annually in more than 150 countries and territories. Approximately 95% of rabies deaths occur in Asia and Africa. China has the second highest number of human rabies deaths, following India [1]. Since the 1950s, three prevalence peaks of human rabies occurred in China. The first prevalence peak appeared in the middle of 1950s, with 1942 deaths due to rabies recorded in 1957. The second prevalence peak occurred in the early 1980s, and 7037 human rabies cases were reported in 1981. The third prevalence peak appeared during the early 21st century, and approximately 3300 cases were reported in 2007 [2]. With the introduction of the ‘One Health’ strategy, control and elimination of rabies were hoped to be achieved by 2030 (WHO, 2016) [3] and some Chinese experts hoped to achieve this goal by 2020. Therefore, more attention should be paid to this neglected disease which continues to impose a heavy burden on developing countries [4].

Although rabies is almost invariably fatal, it can be effectively prevented through appropriate treatment and proper immunisation measures. At present, post-exposure prophylaxis (PEP) with standardised wound treatments is the only method for preventing human rabies after a bite or scratch from an infected animal. Every year, more than 15 million people worldwide receive PEP for rabies [5]. China adopted a 3-dose vaccine schedule for pre-exposure prophylaxis and a 5-dose vaccine schedule for PEP. Individuals with category-III exposures and severely immunocompromised individuals with category-II exposures should receive both rabies vaccines and rabies immunoglobulin (RIG). However, high costs and the self-pay model of mainland China could not guarantee high vaccination rates after exposure. The vaccination schedules used in mainland China include the Essen and Zagreb regimens, which are both recommended by the World Health Organisation. Immunisation procedures are based on the user instructions approved by the China Food and Drug Administration.

In mainland China, the government took effective measures and devoted large amounts of manpower, materials and capital to control rabies. Subsequently, the number of human rabies cases decreased by approximately 84.4% from 2007 to 2017, and only 516 cases were reported in 2017 [6]. Although rabies deaths kept decreasing after 2007, the number of rabies deaths remained among the top three notifiable infectious diseases in mainland China. Among the reported rabies cases, 22% were children and teenagers (younger than 19-years-old), with a mean age of 11 years (data from 2004 to 2014) and most cases were reported in rural areas [7].

Before attempting to understand why some reported cases still died after receiving PEP, it is first necessary to accurately quantitate the PEP vaccination rate among these cases. Other factors such as time elapsed from exposure to treatment, wound care, compliance with vaccination schedules and vaccine quality should also be taken into consideration. Numerous studies have examined immunisation rates among human rabies cases in different areas of China, but no accurate summary data about this proportion is available. For proper planning and implementation of policies designed to achieve elimination of the disease, accurate data about PEP vaccination rates should be obtained. Therefore, the aim of the current study was to estimate the PEP vaccination rate among human rabies cases in mainland China. These data can be used as evidence for policymakers, so that the aim of eliminating human rabies in mainland China by 2020 [8] may be achieved as soon as possible and related health resources can be distributed efficiently.

## Methods

This systematic review followed the Preferred Reporting Items for Systematic Reviews and Meta-Analyses guidelines.

### Search strategy

For the literature search, various Chinese language databases (China National Knowledge Infrastructure, Chinese Science and Technology Periodical Database and Wanfang) and English language databases (PubMed and Web of Science database) were searched for studies on rabies epidemiology that were published between 2007 and 15 December 2017. The search terms used were ‘rabies’, ‘epidemiology or epidemiological or epidemic characteristics’, and ‘China or Chinese’. We obtained additional references from some citations in highly relevant publications but included no data from unpublished studies.

### Selection criteria

Inclusion criteria: (i) Studies with data on morbidity, number of rabies cases and PEP vaccination status of human rabies cases; (ii) studies including rabies patients living in mainland China; and (iii) studies containing data from the Rabies Surveillance Network or community-based survey.

Exclusion criteria: (i) Studies on the epidemiology of swine pseudorabies, (ii) studies that include the risk factors and side effects of vaccine but without vaccination status among rabies cases, (iii) studies without key data on the number of rabies cases or vaccination, (iv) studies with repeated literature or showing repeated results, (v) studies based on hospital data and (vi) studies that showed the vaccination status of the exposed population but without related data among rabies cases.

### Document screening and data extraction

The literature search and data extraction were conducted independently by two researchers (DLW and XQC). They screened the titles and abstracts of studies by consensus and then obtained the full texts of selected citations and assessed the included citations. The extracted characteristics of each citation included the first author, publication year, site of study, sample size, vaccination cases, study time, category-III cases, vaccination status, etc. When a dispute arose, the third researcher (HJ) was asked to reach an agreement. When studies included the same or overlapping data, only the latest data were included in this meta-analysis.

### Document quality evaluation

The included studies were assessed using the Agency for Healthcare Research and Quality (AHRQ) scale that consists of 11 items [9]. The questions in this survey are answerable by ‘yes’ (2 points), ‘no’ (0 point), or ‘do not know’ (1 point). The quality assessment was independently conducted by two researchers (DLW and XQC) and any ambiguity was discussed with the third contributor (XCW).

### Statistical analysis

Excel and R software were used to extract and analyse the data. A meta-analysis of the PEP vaccination rate and its 95% confidence interval (95% CI) were conducted using the metaprop package in R version 3.1.1. In the meta-analysis, PEP vaccination rate was calculated as the ratio of the number of individuals receiving vaccines (full and partial regimens) to the number of rabies deaths. The *χ*^2^ test and *I*^2^ test were used to assess heterogeneity. If there was significant heterogeneity in the data (*I*^2^>50%, *P* < 0.10), the random-effects model was used [10]. Otherwise, the fixed-effects model was used. In a subgroup analysis, the study areas were divided into east, west and central regions according to the ‘method for dividing the east, west, central and northeast regions’ announced by the National Bureau of Statistics in China [11]. The administrative division included province, autonomous region, city, district and county. Subgroup analyses of (i) PEP vaccination rates in different areas, (ii) PEP vaccination rates during different time periods and (iii) different PEP vaccination status before the patient died were conducted. Finally, Begg and Egger's test was performed to analyse publication bias [12, 13].

## Results

### Characteristics of eligible studies

**Fig. 1.**
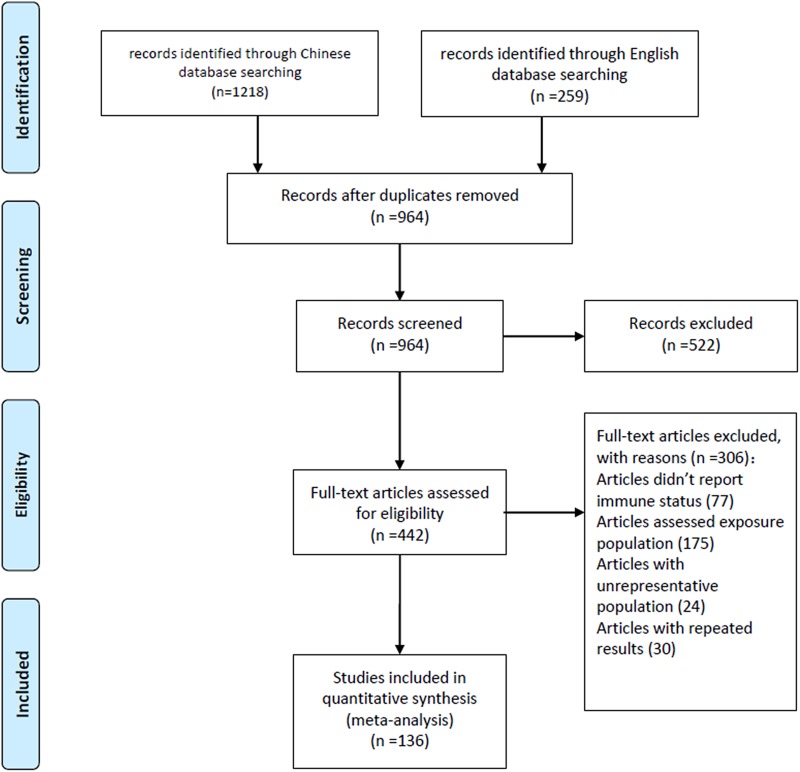
Flowchart of included articles in the meta-analysis.

A total of 1477 articles were initially identified, from which 136 articles were selected for meta-analysis according to the inclusion and exclusion criteria (Fig. 1). The data from the 136 included articles represented 10 174 patients diagnosed with rabies. Most cases were reported from the rabies surveillance network system of the Centres for Disease Control and Prevention. Approximately 97.2% (95% CI 95.1–98.7%) of cases occurred in rural areas，72.6% (95% CI 70.0–75.1%) occurred in farmers and 94.5% (95% CI 93.2–95.8%) resulted from dog bites (Table S1). The characteristics of the included studies are shown in Table S2.

### Random-effects estimate

Both the sample sizes and the PEP vaccination rates reported in the included studies differed greatly; thus, the quality of all studies was considered moderate under the AHRQ scale. *I*^2^ was >50% and the *P* value was <0.1, indicating significant heterogeneity among the included studies. Therefore, a meta-analysis using a random-effects model was conducted. The pooled estimate of PEP vaccination rate among reported rabies cases in mainland China was 15.4% (95% CI 13.7–17.4%) (Fig. S1).

### Publication bias

**Fig. 2.**
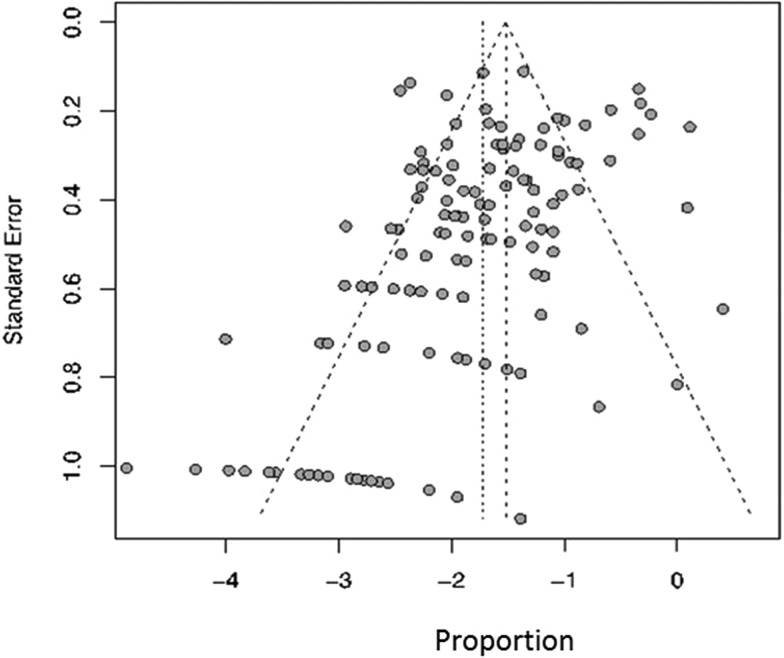
Funnel plots map of PEP vaccination rate.

Funnel plots and distribution plots of 136 articles showed a dispersed distribution. Most of the studies fell in the top side of the funnel plots, but fewer studies were found in the base part and there was some left-right asymmetry, suggesting the possibility of publication bias (Fig. 2).

### Subgroup analysis

Table 1 shows the PEP vaccination rate among human rabies cases stratified by the administration, geography, time duration and vaccination schedule. Due to the high heterogeneity in the subgroups, a random-effects model was used. Under the administrative division, the PEP vaccination rates in the provincial administrative region and the district administrative units were 19.8% (95% CI 10.4–34.3%) and 10.1% (95% CI 6.5–15.4%), respectively. Under the geographic division, the PEP vaccination rate was 18.8% (95% CI 15.9–22.1%) in the eastern region, 13.8% (95% CI 10.0–18.8%) in the central region and 13.3% (95% CI 11.1–15.8%) in the western region. As for time division, the PEP vaccination rate was 19.7% (95% CI 15.3–25.1%) prior to 2007, while it was estimated as 11.3% (95% CI 9.0–14.1%) after 2007. As for vaccination status, individuals receiving full vaccination regimens only accounted for 5.4% (95% CI 4.5–6.4%) of cases, while those receiving partial vaccination accounted for 11.1% (95% CI 9.7–12.5%) of cases.

**Table 1.** Subgroup analysis of PEP vaccination rate among human rabies cases in China

			Vaccination (%)			
Variable		Number of studies	Combined rate	95% CI	*I*^2^	*P*
Total		136	15.4	13.7–17.4	81	<0.01
Administrative division	Province	7	19.8	10.4–34.3	97	<0.01
	City	80	16.8	14.6–19.2	80	<0.01
	District	5	10.1	6.5–15.4	0	0.52
	County	37	13.4	10.6–16.7	47	<0.01
	Autonomous region	6	11.2	7.5–16.3	0	0.44
	Border regions ^a^	1	12.5	5.7–25.2		
Geographical division	East	53	18.8	15·9–22·1	76	<0.01
	Central	31	13.8	10.0–18.8	89	<0.01
	West	51	13.3	11.1–15.8	70	<0.01
	Border regions ^a^	1	12.5	5.7–25.2		
Time division^b^	Before 2007	25	19.7	15.3–25.1	84	<0.01
	Including 2007	93	15.2	13.1–17.6	80	<0.01
	After 2007	18	11.3	9.0–14.1	38	0.05
Vaccination status	Full vaccination	84	5.4	4.5–6.4	44	<0.01
	partial vaccination	84	11.1	9.7–12.5	53	<0.01
	no vaccination	84	83.5	81.6–85.2	54	<0.01

After 2007 = Studies with no data from years up to and including 2007.

a Because the area in this study was a coalition of several border areas, we could not classify it as a region according to the ‘method of dividing the east, west, central and northeast regions’ published by the National Bureau of Statistics in China but identified it as border regions.

b Before 2007 = Studies with no data from 2007 onwards; Including 2007 = Studies with data from 2007.

In this study, individuals with category-III exposures accounted for 68.9% (95% CI 63.6–73.8%) of rabies cases. However, only 10 of the included 136 studies reported the vaccination status of cases with category-III exposures. The pooled estimate of PEP vaccination rate was 27.0% (95% CI 14.4–44.9%) and the rate of RIG administration was 6.1% (95% CI 4.4–8.4%) in individuals with category-III exposures.

Regarding other influencing factors, 11.7% (95% CI 10.1–13.3%) of cases had wounds in the head and face; untreated wounds accounted for 68.9% (95% CI 63.6–73.8%) of rabies cases; self-treated rather than clinic-treated wounds accounted for 22.4%(95% CI 19.3–25.8%) of cases; and vaccination after 24 h accounted for 50.9% (95% CI 18.6–98.3%) of cases.

## Discussion

Rabies remains an important threat to public health in mainland China and it has recently spread to areas where human rabies was previously not reported [2]. Appropriate wound treatment and being fully vaccinated can effectively reduce the incidence of rabies [14]. Recently, Chinese surveillance data showed that the PEP vaccination rate in the exposed population was nearly 96.42%, which resulted in a significant decline in the number of rabies cases in China [15]. However, this meta-analysis indicated that the PEP vaccination rate among reported human rabies cases was only 15.4% (95% CI 13.7–17.4%), which was much lower than the PEP vaccination rate in the exposed population. It meant the low PEP vaccination rate among human rabies cases has, in a large part, led to the tragic outcomes of rabies.

The PEP vaccination rate in different areas, not only at the geographical level but also at the administrative level, differed greatly. The PEP vaccination rate in the western region was substantially lower than that in the eastern region. In mainland China, the economy of the western region is less developed than the eastern region. This result suggested that economy and geographical location were closely related to PEP vaccination rates of rabies. Moreover, limited transportation infrastructure in the western and central areas, coupled with inadequate health resources, made it difficult for people to seek appropriate treatment and timely PEP after exposure. From the perspective of administrative divisions, the PEP vaccination rate at provincial level was somewhat higher because it included data from relatively developed areas. This was similar to a study by Shen *et al*. [16]. After 2007 (the third peak time), the government invested heavily in rabies health education and extensive efforts led to a large decrease in the number of reported human rabies cases. Some surveillance data showed that the proportion of the exposed population receiving a full vaccination regimen was stable at approximately 90% [17–20]. Notably, the PEP vaccination rate among rabies cases decreased over time in this meta-analysis. This finding highlighted that further education is needed to increase the awareness of rabies.

In mainland China, self-funding of PEP vaccination and high vaccination costs were other two factors related to the low vaccination rate. A study by Song *et al*. showed that the total cost for PEP per person, including RIG, nearly reached half of the annual net income of a typical rural resident [21]. As a result, high costs prevented some patients from being vaccinated in a timely and effective manner. Besides, since the PEP vaccination in mainland China is at the patient's discretion, the government cannot confirm the PEP vaccination rate.

Nonstandard PEPs may contribute to PEP failure and subsequent death especially among individuals with category-III exposures. This meta-analysis found that most human rabies cases did not treat their wounds or complete the full courses of vaccination, which significantly contributed to the incidence of rabies [22]. Therefore, education of proper PEP should be enforced. Among individuals with category-III exposures who should receive both an effective vaccine and RIG, the PEP vaccination rate was 27.0% (95% CI 14.4–44.9%). Furthermore, the rate of RIG administration among this high-risk group was only 6.3% (95% CI 6.5–8.7%), which was consistent with the results of several surveillance studies [15, 23]. In mainland China, the low rate of RIG administration could be caused by its high price [24]. In addition, some clinics did not have enough RIG supplies [25]. Consequently, these findings suggested that nonstandard PEPs, such as untreated wounds, partial vaccination, lack of RIG, or restricted access to treatment due to poverty and remoteness, may contribute to PEP failure.

In addition, human rabies frequently occurred in rural residents and children. The absence of rabies awareness among farmers and their children, together with poor medical treatment, made it difficult to eliminate rabies in these rural areas. These imperceptibly formed a malignant cycle. Similarly, a study conducted by Tan *et al*. reported that 65.0% of rabies cases occurred in farmers and 22.0% in children and teenagers under 19 years (from 2004 to 2014) [7]. Therefore, increasing public awareness and strengthening primary medical care in these areas is critical to improve the rabies PEP vaccination rate. Parents decide whether to seek PEP for their children; hence, we should encourage parents to bring their children to medical institutions for treatments and vaccination after exposure, to reduce the risk of rabies in children and teenagers.

There were several limitations to this meta-analysis. The vaccination rate calculated with rabies cases as a denominator in this meta-analysis could not represent the real vaccination rate of the exposed population. Further analysis of the vaccination status of rabies cases may provide clues regarding the reasons for PEP failure. The heterogeneity in the pooled estimates, likely due to different research time and places in included studies, limited the usefulness of the estimates. Besides, data used in most studies were from reported rabies cases and the situation among unreported cases could not be explored. Therefore, this meta-analysis cannot avoid reporting bias. Furthermore, we did not collect the data from unpublished articles and publication bias may have affected the results of our study. Finally, the studies included in the subgroup analysis used various standard case definitions and data of key variables, such as category, vaccination status and treatments of the wound, were found to be inadequate. This introduced bias into our results.

In summary, this meta-analysis showed that the vaccination rate among human rabies cases in mainland China was low. Standardised treatment of dog bites and the awareness of the importance of seeking PEP need to be further strengthened, especially in rural areas. The PEP vaccination rate must be improved in rural areas where people have limited access to appropriate treatment. Enhanced canine vaccination campaigns may also be helpful to eliminate human rabies in mainland China.

## Supplementary material

For supplementary material accompanying this paper visit https://doi.org/10.1017/S0950268818003175. click here to view supplementary material
