# Supplementary material for: Post-exposure prophylaxis vaccination rate and risk factors of human rabies in mainland China: a meta-analysis
Source: Epidemiol Infect. 2018 Dec 4;147:e64. doi: 10.1017/S0950268818003175 (PMC6518593; doi:10.1017/S0950268818003175)
Supplement: Supplementary file 1 [file S0950268818003175sup001.zip › S0950268818003175sup001/[Wang]Supplementary_Table_S1.docx]

**Table S1 The proportion of different groups among human rabies cases in China**

| Group | | Number of studies | | | Proportion | | | |
| --- | --- | --- | --- | --- | --- | --- | --- | --- |
|  |  |  |  |  | Combined proportion（%） | 95%*CI*（%） | *I^2^*（%） | *P* |
| Animal | Dog | | 117 | 94.5 | | 93.2, 95.8 | 85 | <0·01 |
|  | Cat | | 83 | 5.4 | | 4.6, 6.3 | 47 | <0·01 |
| Occupation | Farmer | | 113 | 72.6 | | 70.0, 75.1 | 84 | <0·01 |
|  | Students | | 102 | 14.7 | | 12.8, 16.8 | 80 | <0·01 |
|  | Children | | 71 | 9.9 | | 8.8, 11.2 | 48 | <0·01 |
| Area | Countryside | | 26 | 97.2 | | 95.1, 98.7 | 65 | <0·01 |
|  | City | | 26 | 2.8 | | 1.3, 4.8 | 62 | <0·01 |
