# Supplementary material for: Post-exposure prophylaxis vaccination rate and risk factors of human rabies in mainland China: a meta-analysis
Source: Epidemiol Infect. 2018 Dec 4;147:e64. doi: 10.1017/S0950268818003175 (PMC6518593; doi:10.1017/S0950268818003175)
Supplement: Supplementary file 1 [file S0950268818003175sup001.zip › S0950268818003175sup001/[Wang]Supplementary_Table_S2.docx]

**Table S2: Characteristics of studies identified through systematic review**

| **Studies** | **Date** | **Study location** | **Reported cases** | **Vaccination** | **Category -III cases** | **Rabies immunoglobulin** |
| --- | --- | --- | --- | --- | --- | --- |
| Hu et al (2017)[[1](#_ENREF_1)] | 2010-2014 | Hunan province | 580 | 46 | 462 | 21 |
| Yao et al (2017)[[2](#_ENREF_2)] | 2005-2014 | Jiangyin city | 10 | 3 | 8 | 0 |
| Jiang et al (2016)[[3](#_ENREF_3)] | 2006-2015 | Gaozhou city | 77 | 9 | 48 | - |
| Li et al (2016)[[4](#_ENREF_4)] | 2005-2014 | Zhengzhou city | 64 | 5 | 34 | 1 |
| Zhang et al (2016)[[5](#_ENREF_5)] | 2009-2013 | Shanxi province | 200 | 31 | 186 | 3 |
| Xia et al (2016)[[6](#_ENREF_6)] | 2006-2015 | Dushan county | 54 | 0 | 52 | 0 |
| Wang et al(2016)[[7](#_ENREF_7)] | 2013-2015 | Pingliang county | 10 | 1 | 8 | 0 |
| Lu et al(2016)[[8](#_ENREF_8)] | 2008-2014 | Shijiazhuang city | 91 | 16 | 36 | 0 |
| He et al(2016)[[9](#_ENREF_9)] | 2007-2015 | Huaihua city | 105 | 10 | - | 4 |
| Zhang et al(2016)[[10](#_ENREF_10)] | 2005-2014 | Wanquan county | 6 | 3 | 6 | 0 |
| Jin et al(2016)[[11](#_ENREF_11)] | 2007-2014 | Sheyang county | 8 | 1 | 8 | 0 |
| Wang et al(2016)[[12](#_ENREF_12)] | 2002-2014 | Guangzhou city | 35 | 3 | - | 0 |
| Wu et al(2015)[[13](#_ENREF_13)] | 2005-2014 | Suiyang county | 29 | 0 | 23 | 0 |
| Su et al(2015)[[14](#_ENREF_14)] | 2005-2014 | Liucheng county | 47 | 0 | 37 | 0 |
| Li et al(2015)[[15](#_ENREF_15)] | 2001-2013 | Taizhou city | 99 | 16 | - | - |
| Mao et al(2015)[[16](#_ENREF_16)] | 2008-2013 | Laibin city | 139 | 13 | - | 3 |
| Xu et al(2015)[[17](#_ENREF_17)] | 1987-2013 | Xuchang city | 132 | 0 | 24 | - |
| Liang et al(2015)[[18](#_ENREF_18)] | 2005-2012 | Luoding city | 60 | 3 | - | 0 |
| Zhang et al(2015)[[19](#_ENREF_19)] | 2011-2012 | Ningxia autonomous region | 5 | 1 | 5 | - |
| Li et al(2015)[[20](#_ENREF_20)] | 2000-2014 | Luchuan county | 98 | 23 | - | - |
| Chen et al(2015)[[21](#_ENREF_21)] | 2007-2014 | Hechuan block | 49 | 6 | - | 2 |
| Jia et al(2015)[[22](#_ENREF_22)] | 2000-2013 | Baotou city | 17 | 0 | - | - |
| Wu et al(2015)[[23](#_ENREF_23)] | 2009-2013 | Sandu autonomous region | 14 | 0 | - | - |
| He et al(2015)[[24](#_ENREF_24)] | 2004-2013 | Xingning city | 32 | 8 | 11 | 4 |
| Ren et al(2015)[[25](#_ENREF_25)] | 2007-2014 | Zhejiang province | 201 | 17 | 142 | 4 |
| Chen et al(2014)[[26](#_ENREF_26)] | 2008-2012 | Longhui county | 25 | 1 | 23 | 1 |
| Yang et al(2014)[[27](#_ENREF_27)] | 2002-2013 | Cangwu county | 91 | 18 | - | 2 |
| Liu et al(2014)[[28](#_ENREF_28)] | 2007-2013 | Luoyang city | 35 | 3 | 30 | 0 |
| Xu et al(2014)[[29](#_ENREF_29)] | 1993-2013 | Luzhou city | 49 | 2 | 37 | 0 |
| Bi et al(2014)[[30](#_ENREF_30)] | 2006-2013 | Tai'an city | 50 | 14 | 43 | 0 |
| Liu et al(2014)[[31](#_ENREF_31)] | 2010-2012 | Weinan city | 47 | 7 | 41 | 0 |
| Qiu et al(2014)[[32](#_ENREF_32)] | 2011-2012 | Huizhou city | 23 | 3 | 7 | 0 |
| Wang et al(2014)[[33](#_ENREF_33)] | 2007-2012 | Zhoukou city | 72 | 38 | 64 | 0 |
| Song et al(2014)[[34](#_ENREF_34)] | 2005-2012 | Pingdingshan city | 58 | 11 | - | 2 |
| Yue et al(2014)[[35](#_ENREF_35)] | 2005-2012 | Chengdu city | 95 | 10 | 68 | 2 |
| Pan et al(2013)[[36](#_ENREF_36)] | 2005-2012 | Wuming county | 27 | 3 | 20 | - |
| Qiu et al(2013)[[37](#_ENREF_37)] | 2008-2012 | Qingdao city | 29 | 2 | 22 | 0 |
| Wu et al(2013)[[38](#_ENREF_38)] | 2002-2012 | Chaling county | 72 | 1 | - | - |
| Liu et al(2013)[[39](#_ENREF_39)] | 2000-2011 | Pubei county | 85 | 15 | - | - |
| Huang et al(2013)[[40](#_ENREF_40)] | 1999-2012 | Guilin city | 687 | 59 | 271 | - |
| Wang et al(2013)[[41](#_ENREF_41)] | 2002-2011 | Zhoukou city | 183 | 76 | 169 | 0 |
| Lu et al(2013)[[42](#_ENREF_42)] | 2004-2012 | Qinnan block | 68 | 5 | 31 | 0 |
| Liu et al(2013)[[43](#_ENREF_43)] | 2005-2012 | Yichun city | 77 | 7 | 73 | - |
| Xia et al(2013)[[44](#_ENREF_44)] | 2003-2012 | Xing'an county | 44 | 7 | - | 0 |
| Cheng et al(2013)[[45](#_ENREF_45)] | 2007-2009 | Binzhou city | 26 | 6 | 17 | 0 |
| Ma et al(2013)[[46](#_ENREF_46)] | 2005-2012 | Guanyun county | 17 | 4 | 15 | 0 |
| Wang et al(2013)[[47](#_ENREF_47)] | 2006-2012 | Xintai city | 24 | 6 | 24 | 0 |
| Wu et al(2012)[[48](#_ENREF_48)] | 2008-2011 | Xiangxi autonomous region | 48 | 3 | 30 | 1 |
| Zhao et al(2012)[[49](#_ENREF_49)] | 2006-2010 | Pingdingshan city | 112 | 40 | 59 | - |
| Feng et al(2012)[[50](#_ENREF_50)] | 2010-2011 | Hainan province | 115 | 11 | 65 | 2 |
| Li et al(2012)[[51](#_ENREF_51)] | 2007-2010 | Fengdu county | 46 | 2 | - | 1 |
| Liu et al(2012)[[52](#_ENREF_52)] | 2004-2010 | Nanchang city | 32 | 4 | 30 | 1 |
| Wu et al(2012)[[53](#_ENREF_53)] | 2008-2010 | Honghe autonomous region | 85 | 8 | - | 0 |
| Wu et al(2012)[[54](#_ENREF_54)] | 2006-2010 | Wanzhou block | 36 | 0 | - | 0 |
| Zhou et al(2012)[[55](#_ENREF_55)] | 2006-2010 | Zhuzhou city | 72 | 0 | - | 0 |
| Huang et al(2012)[[56](#_ENREF_56)] | 2001-2010 | Pingyuan county | 16 | 2 | - | 1 |
| Huang et al(2012)[[57](#_ENREF_57)] | 2001-2010 | Jiaojie junction | 48 | 6 | - | 2 |
| Yang et al(2012)[[58](#_ENREF_58)] | 2007-2011 | Peiling block | 37 | 5 | 25 | 0 |
| Liang et al(2012)[[59](#_ENREF_59)] | 2007-2010 | Taishan city | 23 | 1 | 14 | 0 |
| Wang et al(2012)[[60](#_ENREF_60)] | 2004-2010 | Zhengzhou city | 41 | 9 | 29 | 0 |
| Chen et al(2012)[[61](#_ENREF_61)] | 2006-2011 | Xiantao city | 19 | 0 | 4 | 0 |
| Chen et al(2012)[[62](#_ENREF_62)] | 2009-2011 | Xiangyang city | 40 | 3 | - | 0 |
| J.P.Montgomery et al(2012)[[63](#_ENREF_63)] | 2005-2011 | Tianjin city | 60 | 8 | 39 | 1 |
| Yu et al(2011)[[64](#_ENREF_64)] | 2008-2009 | Suizhou city | 32 | 7 | 11 | 0 |
| Xiong et al(2011)[[65](#_ENREF_65)] | 2006-2009 | Shiyan city | 34 | 2 | 24 | 0 |
| Qi et al(2011)[[66](#_ENREF_66)] | 2005-2010 | Xintai city | 20 | 5 | 20 | 0 |
| Zhang et al(2011)[[67](#_ENREF_67)] | 2001-2009 | Pingyue county | 50 | 4 | 1 | 0 |
| Cheng et al(2011)[[68](#_ENREF_68)] | 2004-2010 | Xiangtan city | 91 | 11 | 30 | 3 |
| Jiang et al(2011)[[69](#_ENREF_69)] | 1998-2010 | Nantong city | 34 | 9 | - | 1 |
| Pang et al(2011)[[70](#_ENREF_70)] | 2005-2010 | Jinhua city | 32 | 3 | - | 0 |
| Luo et al(2011)[[71](#_ENREF_71)] | 1987-2009 | Yangshuo county | 56 | 8 | - | 1 |
| Yang et al(2011)[[72](#_ENREF_72)] | 2004-2010 | Zibo city | 10 | 6 | 6 | 0 |
| Zhou et al(2010)[[73](#_ENREF_73)] | 2002-2008 | Ningbo city | 50 | 9 | - | 0 |
| Liang et al(2010)[[74](#_ENREF_74)] | 2002-2009 | Binyang county | 48 | 10 | 39 | 2 |
| Chen et al(2010)[[75](#_ENREF_75)] | 2000-2009 | Lianzhou city | 41 | 4 | - | 0 |
| Jiang et al(2010)[[76](#_ENREF_76)] | 2004-2008 | Changde city | 116 | 10 | 63 | 0 |
| Liu et al(2010)[[77](#_ENREF_77)] | 1999-2008 | Yulin city | 505 | 103 | - | 22 |
| Zhuo et al(2010)[[78](#_ENREF_78)] | 2005-2008 | Xuzhou city | 74 | 17 | 56 | 0 |
| Guo et al(2010)[[79](#_ENREF_79)] | 2003-2009 | Fuyuan county | 23 | 5 | 14 | 1 |
| Qin et al(2010)[[80](#_ENREF_80)] | 2004-2009 | Guilin county | 27 | 1 | - | 0 |
| Ma et al(2010)[[81](#_ENREF_81)] | 2007-2008 | Qionghai city | 13 | 3 | - | 0 |
| Wu et al(2010)[[82](#_ENREF_82)] | 2008-2009 | Yuexi county | 6 | 2 | - | 0 |
| Huang et al(2010)[[83](#_ENREF_83)] | 2004-2009 | Yun'an county | 34 | 2 | - | 0 |
| Tan et al(2010)[[84](#_ENREF_84)] | 2007-2009 | Baise city | 130 | 15 | - | 0 |
| Xu et al(2010)[[85](#_ENREF_85)] | 2003-2004 | Lianyungang city | 58 | 15 | - | - |
| Wang et al(2010)[[86](#_ENREF_86)] | 2004-2009 | Shandong province | 389 | 142 | - | - |
| Peng et al(2009)[[87](#_ENREF_87)] | 2003-2008 | Tianzhu county | 29 | 6 | 21 | 2 |
| Kong et al(2009)[[88](#_ENREF_88)] | 2005-2009 | Zoucheng city | 23 | 12 | 18 | 1 |
| Feng et al(2009)[[89](#_ENREF_89)] | 2003-2008 | Lianyungang city | 83 | 16 | - | - |
| Shi et al(2009)[[90](#_ENREF_90)] | 2002-2007 | Zhumadian city | 104 | 28 | 68 | - |
| Li et al(2009)[[91](#_ENREF_91)] | 2007 | Neimenggu autonomous region | 6 | 2 | 6 | 1 |
| Liu et al(2009)[[92](#_ENREF_92)] | 2005-2007 | An'xi county | 20 | 2 | 7 | 1 |
| Sun et al(2009)[[93](#_ENREF_93)] | 2003-2007 | Tancheng county | 34 | 10 | 9 | 1 |
| Shi et al(2009)[[94](#_ENREF_94)] | 2007-2008 | Mianyang city | 15 | 2 | 13 | 0 |
| Wu et al(2009)[[95](#_ENREF_95)] | 2004-2008 | Gaozhou city | 46 | 6 | - | - |
| Zheng et al(2009)[[96](#_ENREF_96)] | 2001-2007 | Bobai county | 95 | 16 | 67 | 5 |
| Zhou et al(2009)[[97](#_ENREF_97)] | 1999-2007 | Rugao city | 48 | 14 | 40 | 0 |
| Li et al(2009)[[98](#_ENREF_98)] | 2005-2008 | Bobai county | 46 | 5 | - | 1 |
| Bai et al(2009)[[99](#_ENREF_99)] | 2008 | Zhaotong city | 38 | 1 | 38 | 0 |
| Li et al(2009)[[100](#_ENREF_100)] | 2004-2007 | Quzhou city | 13 | 2 | - | 0 |
| Wu et al(2009)[[101](#_ENREF_101)] | 2003-2007 | Beihai city | 62 | 16 | 50 | - |
| Yin et al(2009)[[102](#_ENREF_102)] | 2007 | Chengdu city | 52 | 3 | - | - |
| Wu et al(2008)[[103](#_ENREF_103)] | 2005-2007 | Xiangxi autonomous region | 61 | 8 | 23 | 1 |
| Meng et al(2008)[[104](#_ENREF_104)] | 2004-2006 | Guilin county | 16 | 1 | - | 0 |
| Wang et al(2008)[[105](#_ENREF_105)] | 2003-2007 | Sihong county | 30 | 4 | 15 | 0 |
| Zeng et al(2008)[[106](#_ENREF_106)] | 2006-2007 | Zhaotong city | 39 | 6 | - | 0 |
| Ye et al(2008)[[107](#_ENREF_107)] | 1981-2005 | Cixi city | 32 | 5 | - | 0 |
| Liu et al(2008)[[108](#_ENREF_108)] | 2006 | Songtao county | 15 | 1 | 15 | 0 |
| Li et al(2008)[[109](#_ENREF_109)] | 2007 | Yizhang county | 11 | 2 | 11 | 2 |
| Zhou et al(2008)[[110](#_ENREF_110)] | 2002-2006 | Yongzhou city | 365 | 42 | 265 | 14 |
| Zhou et al(2008)[[111](#_ENREF_111)] | 2007 | Xinjin county | 5 | 0 | - | 0 |
| Ma et al(2008)[[112](#_ENREF_112)] | 1982-2006 | Jiangyin city | 45 | 16 | - | 1 |
| Long et al(2008)[[113](#_ENREF_113)] | 2002-2007 | Fangchenggang city | 61 | 7 | - | 0 |
| Wu et al(2008)[[114](#_ENREF_114)] | 2004-2006 | Ganzhou city | 88 | 27 | - | 0 |
| Liu et al(2008)[[115](#_ENREF_115)] | 2007 | Wusheng county | 16 | 0 | 16 | 0 |
| Wang et al(2008)[[116](#_ENREF_116)] | 2003-2006 | An'qing city | 31 | 5 | - | 2 |
| Liu et al(2008)^)[^[^117^](#_ENREF_117)^]^ | 1997-2007 | Guangzhou city | 44 | 5 | - | 1 |
| Jiang et al(2008)[[118](#_ENREF_118)] | 2006-2007 | Jining city | 49 | 10 | 38 | 0 |
| Yu et al(2008)[[119](#_ENREF_119)] | 2004-2006 | Shangqiu city | 65 | 27 | 48 | 2 |
| Xie et al(2007)[[120](#_ENREF_120)] | 2001-2006 | Meizhou city | 127 | 22 | - | 5 |
| Chen et al(2007)[[121](#_ENREF_121)] | 2001-2005 | Yandu block | 16 | 2 | - | 0 |
| Li et al(2007)[[122](#_ENREF_122)] | 1997-2003 | Zhumadian city | 10 | 2 | - | 1 |
| Xie et al(2007)[[123](#_ENREF_123)] | 1998-2006 | Meixian county | 18 | 4 | 14 | 2 |
| Chen et al(2007)[[124](#_ENREF_124)] | 2001-2006 | Xiangxiang city | 53 | 6 | 7 | 2 |
| Zhang et al(2007)[[125](#_ENREF_125)] | 1974-2005 | Linyi city | 600 | 91 | - | 0 |
| Lu et al(2007)[[126](#_ENREF_126)] | 2004-2006 | Laibin city | 145 | 23 | - | 0 |
| Miu et al(2007)[[127](#_ENREF_127)] | 1983-2006 | Wuxi city | 113 | 29 | - | - |
| Dai et al(2007)[[128](#_ENREF_128)] | 1970-2006 | Yangdong county | 111 | 2 | - | 0 |
| Tan et al(2008)[[129](#_ENREF_129)] | 2003-2007 | Laibin city | 178 | 22 | 159 | 3 |
| Zhou et al(2007)[[130](#_ENREF_130)] | 2000-2004 | Wenzghou city | 26 | 6 | - | 0 |
| Ding et al(2007)[[131](#_ENREF_131)] | 2004-2005 | Shandong province | 124 | 52 | - | 5 |
| Liang et al(2007)[[132](#_ENREF_132)] | 2006 | Renshou county | 18 | 0 | 7 | 0 |
| Ma et al(2007)[[133](#_ENREF_133)] | 2005-2006 | Chengdu city | 27 | 5 | - | 0 |
| Fan et al(2007)[[134](#_ENREF_134)] | 2001-2006 | Jiangyin city | 69 | 11 | 14 | 0 |
| Zhu et al(2007)[[135](#_ENREF_135)] | 2005-2006 | Shengzhou city | 8 | 0 | 8 | 0 |
| Fu et al(2007)[[136](#_ENREF_136)] | 1995-2006 | Yunnan province | 95 | 42 | - | 1 |

Reference

1.**Hu XK, et al.** Epidemiological characteristics of human rabies and failure cases of post-exposure treatment in Hunan Province, 2010-2014. *Practical Preventive Medicine*. 2017;**24**:152-155.

2.**Yao JX, Lu BF, Pan Y**. Epidemiological characteristics of rabies in Jiangyin city of Jiangsu Province, 2005-2014. *Journal of Medical Pest Control.* 2017:114-116.

3.**Jiang JQ**. Analysis on prevalent features of rabies on Gaozhou city, 2006-2015. *Journal of Diseases Monitor & Control*. 2016;**10**:863-864.

4.**Li GW, Chen QG, Wang WM**. Epidemiological analysis of rabies in Zhengzhou City from 2005 to 2014. *Journal of Medical Pest Control.* 2016:914-916.

5.**Zhang Y, Jin Y, Li YJ**. Epidemiological analysis of rabies in Shanxi from 2009 to 2013. *Journal of Medical Pest Control.* 2016:38-41.

6.**Xia JL**. Comparative Analysis on Epidemiological Characteristics of Rabies in Dushan County From 2006 to 2015. *China Health Standard Management.* 2016;**7**:9-11.

7.**Wang ZL, et al.** Epidemic status of rabies and analysis of epidemiological factors in Pingliang city. *Journal of Today Health*. 2016;**15**.

8.**Lu H, et al.** Epidemic characteristics and factors related to rabies in Shijiazhuang,China, from 2008 to 2014. *Chinese Journal of Vector Biology and Control.* 2016;**27**:194-196.

9.**He J**. Epidemiological characteristics and control measures of rabies in Huaihua city, 2007-2015. *China Health Care & Nutrition*. 2016:308-308.

10.**Zhang R, Li SQ, Cui YZ**. Analysis on epidemiological characteristics and control measures of rabies in Wanquan county,2005-2014. *Journal of Medical Pest Control.* 2016:433-434.

11.**Jin JH, Cao HZ**. Analyze of rabies surveillance results in Sheyang county from 2007 to 2014. *Journal of Medical Pest Control.* 2016:1220-1222.

12.**Wang DH, Wang LH, Wu GZ**. Analysis on epidemiological characteristics and control measures of rabies in Guangzhou city,2002-2014. *Journal of Medical Pest Control.* 2016:138-140.

13.**Wu TY, Fan QZ**. Epidemiological Characteristics of Rabies in Suiyang County, Guizhou Province,2005-2014. *Parasitoses and Infectious Diseases.* 2015;**13**:141-142.

14.**Su GY, Wei MQ, Yang M**. The study on epidemiological characteristics of rabies from 2005 to 2014 in Liucheng county. *Popular Science & Technology.* 2015;**17**:74-75.

15.**Li M, He ZS, Liu LC**. Comparative analysis of epidemiological characterics of two epidemic periods of rabies in Taizhou prefecture. *Modern Preventive Medicine*. 2015;**42**:2508-2510.

16.**Mao WC, et al.** Analysis of epidemiological characteristics of rabies in Laibin city from 2008 to 2013. *China Tropical Medicine.* 2015;**15**:60-63.

17.**Xu XH, Zhang QH, Ma YY**. Analysis on Epidemiological Features of Rabies, Xuchang City, 1987-2013. *Preventive Medicine Tribune.* 2015:118-120.

18.**Liang M, Chen YM**. Epidemiological characteristics of rabies in Luoding city, 2005-2012. *Journal of Applied Preventive Medicine.* 2015:249-250.

19.**Zhang M, Li ZM, Li HJ**. Analysis on epidemiological characteristics of rabies in Ningxia, 2011-2012. *Journal of Ningxia Medical University.* 2015;**37**:210-211.

20.**Li CX, Song FC**. Analysis on epidemiological characteristics of rabies in Luchuan county, Guangxi, 2000-2014. *China Rural Health.* 2015:39-39.

21.**Chen L, Tang XF, Liu BL**. Epidemiological characteristics and control measures of rabies in Hechuan area, 2007-2014. *Journal of Diseases Monitor & Control*. 2015;**9**:551-553.

22.**Jia WJ, Jin DH, Xu YP**. Analysis on epidemiological characteristics of rabies in Baotou city, 2000-2013. *Journal of Diseases Monitor & Control*. 2015;**9**:401-402.

23.**Wu FX, Liang QH, Luo JF**. Analysis on epidemiology of rabies in Sandou autonomous county, Guizhou province, 2009-2013. *Medical Science*. 2015:271-271.

24.**He YL, Wen WM**. Epidemiological analysis of rabies in Xingning city from 2004-2013. *Occupation and Health.* 2015;**31**:824-825.

25.**Ren J, et al.** Human rabies in Zhejiang Province, China. *International Journal of Infectious Diseases. 2015;****38****:77-82.*

26.**Chen CJ, Liu TY**. Epidemiological characteristics of rabies in Longhui county, 2008-2012. *Chinese Rural Health Service Administration.* 2014;**34**:179-180.

27.**Yang MS**. Epidemiological characteristics of rabies in Cangwu county, 2002-2013. *World Latest Medicine Information*. 2014:327-328.

28.**Liu Y, Zhu X**. Study on Epidemiological Characteristics of Rabies and Medical Innovation in Luoyang City. *Medical Innovation of China.* 2014:95-96.

29.**Xu J, Zheng HS, Chang YH**. Epidemiological Characteristics of Rabies and Prevention and Control Countermeasures in Luzhou, 1993-2013. *Journal of Preventive Medicine Information.* 2014;**30**:749-752.

30.Bi XJ, Yin SS, Zhang AH, editors. Analysis of epidemiology and clinic surveillance data of rabies in Tai'an city, 2006-2013. *Academic annual meeting of Shandong association for science and technology in 2014*; 2014.

31.**Liu W, Huo DC, Liu G**. Epidemiological Analysis on Rabies in Weinan District from 2010 to 2012. *Henan Journal of Preventive Medicine.* 2014;**25**:423-426.

32.**Qiu WQ, Liu XM, Ju XF**. Analysis of surveillance data of rabies in Huizhou city, 2011—2012. *South China Journal of Preventive Medicine.* 2014:264-266.

33.**Wang XS**. Epidemiological analysis of rabies in Zhoukou city, 2007-2012. *Preventive Medicine Tribune.* 2014:64-65.

34.**Song QQ, Wang XQ**. Epidemiological analysis of rabies in Pingdingshan city from 2005 to 2012. *China Tropical Medicine.* 2014;**14**:170-172.

35.**Yue Y, et al.** Analysis of epidemiological characteristics on 95 rabies cases in Chengdu city. *Journal of Tropical Diseases and Parasitology.* 2014;**12**:26-28.

36.**Pan YZ, et al.** Analysis on epidemic situation and control measures of rabies in Wuming County from 2005 to 2012. *Journal of Medical Pest Control*. 2013:1083-1085.

37.**Qiu B, Jiang FC**. Epidemiological characteristics of human rabies in Qindao, China from 2008 to 2012. *Chinese Journal of Vector Biology and Control.* 2013;**24**:546-548.

38.**Wu XH**. Analysis of rabies epidemic situation in Chaling County, Hunan Province, 2002-2012. *For all Health.* 2013;**7**:17-18.

39.**Liu X, et al.** Analysis of epidemiological characteristics of rabies in Pubei county from 2000-2011. *Medical Journal of Chinese People's Health.* 2013;**25**:32-34.

40.**X HS, Zhang ZK, Feng HB**. Epidemic Characteristics of Rabies in Guilin, 1999-2012. *Parasitoses and Infectious Diseases*. 2013;**11**:124-126.

41.**Wang FZ**. The analysis to epidemic factors of rabies in Zhoukou city during 2002-2011. *Henan Journal of Preventive Medicine*. 2013;**24**:472-475.

42.**Lu YP, et al.** Epidemic Characteristics of Rabies in Guilin, 1999-2012. *Journal of Applied Preventive Medicine*. 2013;**19**:343-346.

43.**Liu LH, Chen JG, Yang J**. Epidemiological characteristics and control measures of rabies in Yichun city. *Jiangxi Medical Journal*. 2013;**48**:359-368.

44.**Xia HM**. Analysis of rabies epidemic situation in Xingan County, 2003-2012. *Journal of Applied Preventive Medicine.* 2013:100-101.

45.**Cheng ZK, Dai ZH, Yan W**. Investigation and analysis on 26 cases of human rabies in Binzhou. *Journal of Medical Pest Control*. 2013:529-531.

46.**Ma SH**. Epidemiological characteristics of rabies in Guanyun county, 2005-2012. *Jiangsu Journal of Preventive Medicine*. 2013;**24**:66-67.

47.**Wang RH, An BL, Xu LZ**. Analysis of epidemiological characteristics in Xintai city, 2006-2012. *Preventive Medicine Tribune*. 2013:144-145.

48.**Wu G, et al.** Analysis on Surveillance of Rabies in Xiangxi Autonomous Prefeture from 2008 to 2011. *Practical Preventive Medicine*. 2012;**19**:855-857.

49.**Zhao KN**. Analysis on epidemiological situation of rabies in Pingdingshan city. *The Medical Forum*. 2012;**16**:1344-1345.

50.**Feng FL, Jin YM, Jia PB**. Analysis of epidemic characteristics of rabies in Hainan province from 2010 to 2011. *China Tropical Medicine*. 2012;**12**:1330-1332.

51.**Li XB**. Study on epidemiological analysis and prevention measures of rabies, 2007-2010. *Chongqing Medicine.* 2012;**41**:1984-1985.

52.Liu LH. Analysis of epidemic status and control measures of rabies in Nanchang city. *Journal of Medical Pest Control*. 2012:955-957.

53.**Wu GS, Mou JC, Li F**. Epidemiological analysis of rabies in Honghezhou,2008-2010. *Soft Science of Health*. 2012;**26**:417-419.

54.**Wu B, Mao DQ, Yan CY**. Epidemicological analysis and control measures of rabies from 2006 to 2010 in Wanzhou of Chongqing. *Journal of Medical Pest Control*. 2012:93-95.

55.**Zhou LP, Li KX**. Epidemiological analysis of rabies in Zhuzhou city,2006-2010. *Preventive Medicine Tribune.* 2012:73-74.

56.**Huang LJ, et al.** Epidemiological analysis of rabies in Pingyuan, 2001-2010. *Journal of Tropical Medicine*. 2012;**12**:1146-1147.

57.**Huang LJ, Zhang XL, Gu QF**. Rabies Epidemic Situation Analysis of the Junction of Guangdong, Fujian and Jiangxi Procinces in 2001-2010. *Journal of Tropical Medicine*. 2012;**12**:1382-1384.

58.**Yang DM, Ju DH**. Epidemiological characteristics and control measures of rabies in Peilin, 2007-2011. *Journal of Occupational Health and Damage.* 2012;**27**:368-370.

59.**Liang HZ, Liu WJ, Li L**. Epidemiological analysis of rabies in Taishan city, 2007-2010. *Preventive Medicine Tribune.* 2012:237-238.

60.**Wang WM, Gao LH, Chen YZ**. Analysis on the epidemiological characteristics of rabies from 2004 to 2010 in Zhengzhou. *Journal of Medical Pest Control*. 2012:294-295.

61.**Chen L, Liu TJ**. Epidemiological analysis and prevention strategies of rabies in Xiantao city from 2006-2011. *Occupation and Health*. 2012;**28**:2920-2921.

62.**Chen Y, Qin MS, Liu F**. Surveillance status of rabies in Xiangyang city, 2009-2011. *Medical Information.* 2012;**25**:95-96.

63.**Montgomery JP, et al.** Human rabies in Tianjin, China. *Journal of Public Health*. 2012;**34**:505.

64.**Yu JJ, Hu CM**. Epidemiological analysis of 32 rabies cases. *World Health Digest*. 2011;**08**.

65.**Xiong XH, Shen H, Xiao DC**. Epidemiological characteristics of rabies in Shiyan from 2006 to 2009. *Modern Preventive Medicine.* 2011;**38**:821-822.

66.**Qi YX, Su YW, Wu YJ**. Analysis of the epidemic of rabies in Xintai city, 2005-2010. *Preventive Medicine Tribune.* 2011:858-859.

67.**Zhang BJ, Lu HM**. Epidemiological analysis of rabies in Pingle county, 2001-2009. *Journal of Medical Pest Control.* 2011:268-269.

68.**Cheng LZ, et al.** Epidemiological study of rabies in Xiangtan, Hunan from 2004 to 2010. *Practical Preventive Medicine.* 2011;**18**:791-794.

69.**Jiang J**. Epidemiological analysis of rabies in Tongzhou, Nantong from 1998 to 2010. *Journal of Medical Pest Control*. 2011;**27**:847-847.

70.**Pang ZF, JIN ZP, Zhang l**. Epidemiological characteristics of rabies in Jinhua, Zhejiang from 1950 to 2010. *Chinese Journal of Vector Biology and Control*. 2011;**22**:277-279.

71.**Luo JM, Gao ZY, Mo JM**. Epidemic situation of rabies in Yangshuo, Guangxi. *Journal of Tropical Medicine.* 2011;**11**:215-216.

72.**Yang SH, Liu L, Gao YX**. Analysis on data of rabies in Zibo city, 2004-2010. *Chinese Journal of Disease Control & Prevention.* 2011;**15**:1091-1092.

73.**Zhou AM**. Epidemiological analysis of rabies in Ningbo city, 2002-2008. *Preventive Medicine.* 2010;**22**:33-34.

74.**Liang H, et al.** Epidemiological analysis of rabies in Binyang county, Guangxi from 2002-2009. *China Tropical Medicine.* 2010;**10**:1354-1355.

75.**Chen ZB, et al.** Epidemiology of human rabies in Lianzhou city, Guangdong province, 2000-2009. *Disease Surveillance*. 2010;**25**:969-971.

76.**Jiang XW, et al.** Epidemiological survey of rabies in Changde city in 2004-2008. *China Tropical Medicine.* 2010;**10**:37-38.

77.**Liu YW, et al**., editors. Epidemiological survey of 505 rabies cases in Yulin city, Guangxi autonomous region. *Disease Surveillance*. 2010:757-760.

78.**Zhuo W, Zhu QJ**. Epidemiological analysis of rabies in Xuzhou city, 2005-2008. *Chinese Journal of School Doctor.* 2010;**24**:738-738.

79.**Guo JM**. Analysis on epidemiological characteristics of rabies in Fuyuan county of Yunnan province during 2003-2009. *Occupation and Health.* 2010;**26**:2653-2655.

80.**Qin GX**. Epidemiological analysis of rabies in Guilin county, 2004-2009. *Journal of Applied Preventive Medicine.* 2010;**16**:67-68.

81.**Ma T, et al.** Epidemiological analysis of rabies in Qionghai city, 2007-2008. *Modern Preventive Medicine*. 2010;**37**:4340-4340.

82.**Wu ZQ**. Epidemiological characteristics and controlling effect of rabies in Yuexi county, 2008-2009. *Chinese Journal of Clinical Rational Drug Use.* 2010;**03**:137-138.

83.**Huang MA**. Epidemiological analysis of rabies in Yun'an county of Guangdong province during 2004—2009. *Occupation and Health.* 2010;**26**:1964-1966.

84.**Tan Y, Meng ZM, Tang YJ**. Epidemiological characteristics of rabies from 2007 to 2009 and the problems in preventing rabies. *China Animal Health Inspection*. 2010:39-41.

85.**Xu L, Ying L, Chen XL**. Epidemiological Analysis of 58 rabies cases in Lianyungang city. *Occupation and Health.* 2010;**26**:429-430.

86.**Wang X, et al.** Human rabies epidemiology in Shandong Province, China. *Japanese Journal of Infectious Diseases*. 2010;**63**:323.

87.**Peng DJ**. Epidemiological analysis of rabies in Tianzhu county, 2003-2008. *Journal of Preventive Medicine Information*. 2009;**25**:258-260.

88.Kong DY, Xie RF, editors. Epidemiological analysis of rabies in Zoucheng city. *Chinese Journal of Public Health Management*. 2009: 2005-2009.

89.**Feng MY**. Analysis of epidemiological characteristics in one place of Jiangsu province. Chinese *Journal of Ethnomedicine and Ethnopharmacy.* 2009;**18**:87-88.

90.**Shi K, Zhang ZW, Feng JP**. Study on epidemiological characteristics of rabies in Zhumadian area in 2002-2007. *Modern Preventive Medicine.* 2009;**36**:3771-3773.

91.**Li TH, Yang SC, Wang LH**. Analysis of epidemiological characteristics of rabies in Neimenggu, 2007. *Journal of Medical Pest Control.* 2009:121-121.

92.**Liu JB**. Epidemiological analysis of rabies in Anxi county, 2005-2007. *Chinese Journal of Public Health Management.* 2009:210-211.

93.**Sun L, Song YS, Li AL**. Data analysis of rabies in Tancheng county from 2003 to 2007. *Preventive Medicine Tribune*. 2009:364-365.

94.**Shi YH, Liu CD, Huang C**. The epidemiology of rabies and the control measures in the earthquake area of Mianyang district. *Chinese Journal of Zoonoses*. 2009;**25**:1025-1026.

95.**Wu XL, Zhang Y, He YB**. Analysis of the epidemiological characyeristics of rabies in Gaozhou city from 2004 to 2008. *Journal of Medical Pest Control.* 2009:494-496.

96.**Zheng ZG, Li YB, Li YH**. Epidemiological analysis of rabies in Bobai county,2001-2007. *South China Journal of Preventive Medicine*. 2009:46-47.

97.**Zhou GX, You YM**. Epidemiological analysis of rabies in Rugao city, 1999-2007. *Jiangsu Journal of Preventive Medicine*. 2009;**20**:31-32.

98.**Li YP, Chen JX, Mo QB**. Prevalent features of rabies in Bobai county from 2005 to 2008. *China Tropical Medicine*. 2009;**9**:1328-1329.

99.**Bai ZK, Zeng YH, Jin BR**. Epidemiological analysis on 38 rabies cases in Zhaotong, Yunnan, 2008. *Disease Surveillance*. 2009;**24**:764-765.

100.**Li JJ, Yu ZY**. Epidemic status of rabies in Quzhou city, 2004-2007. *Preventive Medicine.* 2009;**21**:22-23.

101.**Wu DR, Xie P, Shen ZY**. Epidemiological analysis of rabies in Beihai city, 2003-2007. *Journal of Applied Preventive Medicine*. 2009;**15**:216-218.

102.**Yin ZL, Yue Y**. Epidemiological analysis of 52 rabies cases in Chengdu in 2007. *Journal of Preventive Medicine Information*. 2009;**25**:293-295.

103.**Wu DK, Qu BY, Huang H**. Analysis of rabies epidemic situation in Xiangxi autonomous prefecture from 2005 to 2007. *Preventive Medicine Tribune.* 2008;**14**:906-907.

104.**Meng XM**. Analysis of prevalent features of rabies in Lingui county in 2004-2006. *China Tropical Medicine.* 2008;**8**:273-274.

105.**Wang YL, Xu BZ, Peng F**. Analysis of rabies epidemic situation in Sihong county, 2003-2007. *Practical Preventive Medicine*. 2008;**15**:449-451.

106.**Zeng YH, et al.** Analysis of epidemiological characteristics of rabies in Zhaotong city, 2006-2007. *Chinese Journal of Disease Control & Prevention*. 2008;**12**:635-636.

107.**Ye JJ, Shi NF, Chen GH**. Analysis on epidemiological characteristics of rabies in Cixi city, 1981-2005. *Preventive Medicine*. 2008;**20**:32-33.

108.**Liu ZX**. Epidemic survey of 15 rabies cases in Songtao county. *Journal of Medical Pest Control*. 2008;**24**:283-284.

109.**Li CH, Hu ZG**. Data analysis of rabies epidemiology in Yizhang county in 2007. *Practical Preventive Medicine.* 2008;**15**:462-463.

110.**Zhou XQ, Yang WL, Xiao J**. Epidemiological analysis of rabies in Yongzhou city, 2002-2006. *South China Journal of Preventive Medicine.* 2008;**34**:43-44.

111.**Zhou HQ**. Analysis on rabies epidemic status in Xinjin county in 2007. *Journal of Preventive Medicine Information.* 2008;**24**:565-565.

112.**Ma Y**. Epidemiological characteristics and control measures of rabies in Jiangyin city, 1982-2006. *Journal of Medical Pest Control*. 2008;**24**:120-121.

113.**Long HY, Huang DF, Xu WJ**. Epidemiological survey of rabies in Fangchenggang city in 2002-2007. *China Tropical Medicine*. 2008;**8**:997-998.

114.**Wu ZF, Liao Y**. Ananlysis of epidemiological situation and surveillance of rabies in Ganzhou. *Modern Preventive Medicine*. 2008;**35**:2139-2141.

115.**Liu JY, Liao PJ**. Epidemiological analysis of rabies in Wusheng county in 2007. *Journal of Preventive Medicine Information.* 2008;**24**:829-830.

116.**Wang JS, Xie J, Shu XP**. Analysis of epidemic features and factors of human rabies from 1953 to 2006 in Anqing city. *Anhui Journal of Preventive Medicine*. 2008:257-259.

117.**Liu XN, et al.** Epidemiological Characteristics and Preventive Strategies of Rabies from 1997 to 2007 in Guangzhou City. *Journal of Tropical Medicine.* 2008;**8**:1272-1274.

118.**Jiang WG, Yan BX**. Analysis of epidemiological characteristics and risk factors for rabies in Jining city from 2006 to 2007. *Journal of Pathogen Biology.* 2008;**3**:619-620.

119.**Yu RF, Wang WM, Chen GS**. Epidemiological analysis of 65 rabies cases in Shangqiu city, 2004-2006. *Henan Journal of Preventive Medicine*. 2008;**19**:204-205.

120.**Xie KQ, Lin LX, Yang YL**. Epidemical analysis of rabies in Meizhou city from 2001 to 2006. *Journal of Tropical Medicine.* 2007;**6**:259-259.

121.**Chen WG, Yang SH, Zhang YX**. Data analysis of rabies in Yandu, Yancheng from 2001 to 2005. *Preventive Medicine Tribune*. 2007;**13**:568-568.

122.**Li XJ, Su GZ**. Epidemiological analysis of rabies in Zhumadian city,1997-2003. *Henan Journal of Preventive Medicine*. 2007;**18**:369-370.

123.**Xie XQ, et al.** Epidemiological survey on rabies in Mei county, 1998-2006. *China Tropical Medicine*. 2007;**7**:786-786.

124.**Chen WN, Fu RH, Liu FQ**. Epidemic situations of human rabies from 2001 to 2006 and its preventive measures in Xiangxiang city. *Practical Preventive Medicine*. 2007;**14**:1450-1452.

125.**Zhang SH, Feng QR, Zuo HB**. Epidemiological analysis of rabies in Linyi city, 1974-2005. *Occupation and Health*. 2007;**23**:1618-1619.

126.**Lu WZ**. Epidemiological analysis of rabies in Laibin city,Guangxi from 2004 to 2006. *China Medical Herald.* 2007;**4**:117-118.

127.**Liao XL**. Epidemiological analysis of human rabies in Wuxi city. *Jiangsu Journal of Preventive Medicine.* 2007;**18**:20-21.

128.**Dai YM, Chen HZ, Chen W**. The Epidemiology Analysis of Rabies in Yangdong from 1970 to 2006. *International Medicine and Health Guidance News*. 2007;**13**:111-114.

129.**Tan SN, et al.** Analysis of prevalence of rabies in Laibin city of Guangxi in 2003～2007. *China Tropical Medicine*. 2008;**8**:989-990.

130.**Zhou ZM, Zhang XC, Jia LT**. Epidemiological analysis of rabies in Wenzhou city of Zhejiang province from 2000 to 2004. *Disease Surveillance*. 2007;**22**:335-337.

131.**Ding SJ, Li Z, Kou ZQ**. Epidemiological analysis of hydrophobia epidemic situation from 2004 to 2005 in Shandong province. *Modern Preventive Medicine*. 2007;**34**:1684-1685.

132.**Liang XW, et al.** Epidemiological investigation on rabies cases and exposed people. *Journal of Preventive Medicine Information*. 2007;**23**:709-711.

133.**Ma L, Wu DB, Hu Q**. Epidemiological characteristics and preventive strategies of rabies from 1950 to 2006 in Chengdu city. *Disease Surveillance*. 2007;**22**:468-470.

134.**Fan ZF, et al.** Epidemic analysis of rabies in Yangjiang city from 2001 to 2006. *South China Journal of Preventive Medicine*. 2007;**33**:34-35.

135.**Zhu XC, Yang ZX, Shi ZW**. Analysis of infectious status of rabies in Chengzhou city and control measures. *China Tropical Medicine*. 2007;**7**:1187-1187.

136.**Fu XQ, Chang LT**. Epidemic situation and control measures of rabies in Yunnan province from 1995 to 2006. *Disease Surveillance*. 2007;**22**:659-661.
