# Supplementary figures and images for: Post-exposure prophylaxis vaccination rate and risk factors of human rabies in mainland China: a meta-analysis
Source: Epidemiol Infect. 2018 Dec 4;147:e64. doi: 10.1017/S0950268818003175 (PMC6518593; doi:10.1017/S0950268818003175)

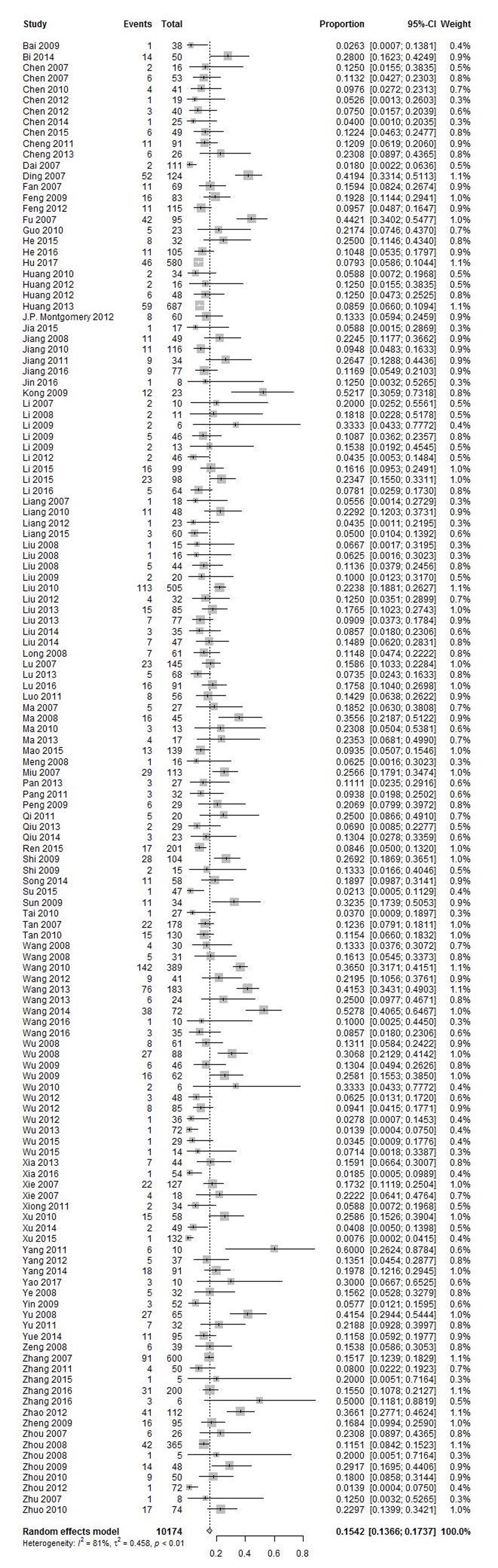

Supplement: Supplementary file 1 [file S0950268818003175sup001.zip › S0950268818003175sup001/[Wang]FigS1.tif]
